# Alterations of brain activity and functional connectivity in transition from acute to chronic tinnitus

**DOI:** 10.1002/hbm.25238

**Published:** 2020-10-13

**Authors:** Liping Lan, Jiahong Li, Yanhong Chen, Wan Chen, Wenrui Li, Fei Zhao, Guisheng Chen, Jiahao Liu, Yuchen Chen, Yuanqing Li, Chang‐Dong Wang, Yiqing Zheng, Yuexin Cai

**Affiliations:** ^1^ Department of Otolaryngology Sun Yat‐sen Memorial Hospital, Sun Yat‐sen University Guangzhou City Guangdong Province China; ^2^ Institute of Hearing and Speech‐Language Science Sun Yat‐sen University Guangzhou City Guangdong Province China; ^3^ State Key Laboratory of Ophthalmology, Zhongshan Ophthalmic Center Sun Yat‐sen University Guangzhou China; ^4^ Department of Speech and Language Therapy and Hearing Science Cardiff Metropolitan University Cardiff UK; ^5^ Department of Hearing and Speech Science, Xinhua College Sun Yat‐Sen University Guangzhou China; ^6^ Department of Radiology Nanjing First Hospital, Nanjing Medical University Nanjing China; ^7^ School of Automation Science and Engineering South China University of Technology Guangzhou China; ^8^ School of Data and Computer Science Sun Yat‐sen University Guangzhou China

**Keywords:** acute tinnitus, chronic tinnitus, local neural activity, multifunctional brain network, transition

## Abstract

The objective of this study was to investigate alterations to brain activity and functional connectivity in patients with tinnitus, exploring neural features in the transition from acute to chronic phantom perception. Twenty‐four patients with acute tinnitus, 23 patients with chronic tinnitus, and 32 healthy controls were recruited. High‐density electroencephalography (EEG) was used to explore changes in brain areas and functional connectivity in different groups. When compared with healthy subjects, acute tinnitus patients had a significant reduction in superior frontal cortex activity across all frequency bands, whereas chronic tinnitus patients had a significant reduction in the superior frontal cortex at beta 3 and gamma frequency bands as well as a significant increase in the inferior frontal cortex at delta‐band and superior temporal cortex at alpha 1 frequency band. When compared to the chronic tinnitus group, the acute tinnitus group activity was significantly increased in the middle frontal and parietal gyrus at the gamma‐band. Functional connectivity analysis showed that the chronic tinnitus group had increased connections between the parahippocampus gyrus, posterior cingulate cortex, and precuneus when compared with the healthy group. Alterations of local brain activity and connections between the parahippocampus gyrus and other nonauditory areas appeared in the transition from acute to chronic tinnitus. This indicates that the appearance and development of tinnitus is a dynamic process involving aberrant local neural activity and abnormal connectivity in multifunctional brain networks.

## Graphical abstract

Our findings suggested that the appearance and development of tinnitus is a dynamic process involving aberrant local neural activity and abnormal connectivity in multifunctional brain networks. The alterations of local brain activity and connections between the parahippocampus gyrus and other nonauditory areas appeared in the transition from acute to chronic tinnitus, which appear important to identify the neural predictors for the development of tinnitus and prevention of transition from acute to chronic tinnitus.

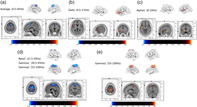

## 1 INTRODUCTION

Tinnitus is the perception of a phantom sound lacking any external or internal sound stimulation. As a common clinical symptom tinnitus is experienced constantly by 10–15% of the adult population (Langguth, Kreuzer, Kleinjung, & De, 2013). It is generally believed that tinnitus is not only the result of a cochlear lesion, but also a symptom involving plasticity of the central nervous system (Chen et al., 2016; Lanting, De, & Van, 2009; Loo et al., 2009). Recent studies have found that abnormal central reorganization in subjective chronic tinnitus involves auditory and nonauditory brain areas such as the frontal cortex, parahippocampal gyrus, caudate nucleus, cingulate cortex, and insula (Araneda et al., 2018; Besteher et al., 2019; De et al., 2014; Elgoyhen, Langguth, Ridder, & Vanneste, 2015; Henderson‐Sabes et al., 2019; Schlee et al., 2009; Vanneste, Alsalman, & De Ridder, 2018; Xu et al., 2019), and that these central neural changes were possible contributors to the clinical symptoms of tinnitus, for example, impairment of perception, cognition, attention, memory, and emotion (Besteher et al., 2019; Chen et al., 2017; De, Elgoyhen, Romo, & Langguth, 2011; Husain, 2016; Joos, Vanneste, & De Ridder, 2012; Pattyn et al., 2016; Vanneste, To, & De Ridder, 2019). For example, Schlee et al. (Schlee et al., 2009) found that abnormal activation of the frontal cortex was related to a conscious perception of subjective chronic tinnitus and Vanneste et al. (Vanneste & De Ridder, 2016) found that the parahippocampal area was significantly activated in chronic tinnitus patients with severe hearing loss. In addition, a positive correlation was found between neural activity in the parahippocampal area and averaged hearing thresholds. As the parahippocampal area has been hypothesized to play a crucial role in memory recollection, sending information from the hippocampus to the association areas, such a correlation might indicate its involvement in the generation of tinnitus.

Chen et al. (Chen et al., 2017) investigated aberrant amygdala‐cortical functional connectivity using a seed‐based whole‐brain correlation method in chronic tinnitus patients with depression and found abnormal resting‐state functional connectivity between the amygdala and prefrontal‐cingulate‐temporal circuit. These aberrant functional couplings may explain the depressive symptoms of chronic tinnitus. A review by Pattyn et al. (Pattyn et al., 2016) concluded that interactions between auditory networks and the limbic system also appeared in tinnitus patients with anxiety and depression. Chen et al. (Chen et al., 2018) also found abnormal connections between the anterior cingulate cortex (ACC) and auditory cortex, prefrontal cortex, visual cortex, and default mode network in chronic tinnitus patients. These abnormally increased connections between the rostral ACC and left precuneus as well as dorsal ACC and right inferior parietal lobe were positively correlated with Tinnitus Handicap Questionnaire (THQ) scores. The abnormal connectivity of ACC and other cortical regions may contribute to tinnitus induced depression.

Although a number of previous studies on chronic tinnitus have been published and many pathophysiological models have been developed, few studies have investigated central plasticity in acute tinnitus (Cai et al., 2020; Stolzberg et al., 2013), especially the dynamic changes to neural processing during transition from acute to chronic tinnitus. This issue is crucial to clarifying the central neural characteristics of acute and chronic tinnitus, which is important in identifying neural predictors for the development of tinnitus and prevention of transition from acute to chronic tinnitus.

A study by Vanneste et al. (Vanneste, van de Heyning, & De Ridder, 2011) aimed to identify neural generator or neural network change over time with tinnitus by comparing EEG responses in chronic tinnitus patients with different durations (< 4 years and > 4 years). Differences were found in auditory and nonauditory areas (anterior cingulate, insula and medial premotor cortex). In the present study, we aimed to explore neural generator and dynamic development of tinnitus by comparing tinnitus patients between acute (< 1 months) and chronic stages (> 6 months). A number of different functional techniques have been used to study central processing in tinnitus; electroencephalography (EEG), functional magnetic resonance imaging (fMRI), magnetoencephalography (MEG), and positron emission tomography (PET). Each approach has its strengths and weakness. For example, fMRI is known to have good spatial resolution. However, fMRI produces aversive scanner noise which interferes with auditory processing in the human brain. The resting state neural response in tinnitus patients can be affected by the fMRI scan because central neural processing is activated by scanner noise even when ear plugs, or headphones with active noise reduction are worn (Logothetis et al., 2009). EEG has the advantage of high temporal resolution, time efficiency, and noise avoidance, and is widely used in source localization and functional connectivity analysis (Van Diessen et al., 2015).

Therefore, to verify the hypothesis and explore the underlying mechanism of subjective tinnitus, this study investigated aberrant central changes between acute and chronic tinnitus patients and healthy controls using resting‐state EEG. These finding may be important to identify the central processing network and neural predictors for the development of tinnitus.

## 2 METHODS

### 2.1 Participants

Twenty‐four patients with acute tinnitus (duration less than 1 month) and 23 patients with chronic tinnitus (duration more than 6 months) were recruited from the Ear, Nose and Throat clinic, Sun Yat‐sen Memorial Hospital, Sun Yat‐sen University.

The selection criteria for subjects with tinnitus were:

- Patients had sought clinical help for their tinnitus.
- Patients with conductive or mixed hearing loss were excluded
- Patients had no history of use of ototoxic drugs
- Subjects with middle ear surgery, pulsatile tinnitus, Ménière's disease, autoimmune hearing loss, acoustic neurinoma, central nervous system disorders, and head trauma were excluded.

Thirty‐two healthy controls without tinnitus were also recruited. None of participants was reported comorbid conditions alongside tinnitus such as hypertension, hyperacusis, depression, epilepsy. Acute tinnitus patient data were collected prior to receiving any therapeutic intervention. All participants were provided with information about this study and signed a written consent form. This research was approved by the Institution Review Board of The Sun Yat‐sen Memorial Hospital.

### 2.2 Audiological investigations

All participants were interviewed to obtain a thorough case history including; age, duration of tinnitus, side of tinnitus and any other pathologies. This was followed by otoscopic examination, pure tone audiometry and a tinnitus matching test. The hearing threshold was determined using pure tone audiometry: air conduction hearing thresholds were measured at frequencies between 125 and 8,000 Hz with an octave interval. In addition, bone conduction hearing thresholds were measured at 250, 500, 1,000, 2000, and 4,000 Hz. Mean hearing threshold was calculated as the average of hearing thresholds at 500, 1000, 2000, and 4,000 Hz (Bing et al., 2018; Dispenza et al., 2011).

### 2.3 Tinnitus pitch and loudness matching tests

At first, nine audiometric frequencies between 125 and 8.0 kHz (125, 250, 500 Hz and 1.0, 2.0, 3.0, 4.0, 6.0, and 8.0 kHz) were used to approximately match the tinnitus pitch. Subjects were asked to compare their perceived tinnitus pitch with different matching tones until the tone exactly matching their tinnitus was obtained. If no pure tone was obtained, narrowband noise was used. After confirming tinnitus pitch, the level was set 5 dB above the measured audiometric threshold to give an approximate tinnitus loudness level, which was then adjusted in 1 dB step until the subject reported that the tone matched the loudness of their tinnitus (Kim et al., 2016).

### 2.4 Tinnitus handicap inventory

A tinnitus handicap inventory (THI) was used to evaluate tinnitus severity and level of tinnitus handicap, according to 0–100 increasing handicap scale. Participants completed the THI prior to the experiment.

### 2.5 EEG data collection

A high‐density EEG with 128channels (EGI, Eugene) and a NetAmps 200 amplifier was applied to collect resting‐state EEG data from all participants. Subjects were instructed to sit in a comfortable chair and remain calm. Subjects were then required to keep their eyes open and fixate a cross mark in front of them. The EEG recording lasted ~7 min. The CZ electrode was used as reference. The sampling rate was 1,000 Hz. Impedances were kept to less than 50 kΩ.

### 2.6 Preprocessing of EEG data

EEGLAB for v13.0.0 toolbox in MATLAB for R2013a was used to preprocess the raw EEG data. Firstly, the data were re‐referenced against the mean reference for all electrodes. The sampling rate was adjusted to 0.5 kHz. A notch filter was implemented at 50 Hz and the signals were band‐pass‐filtered from 0.5 to 100 Hz. Gross artifacts were manually removed by visual inspection. Artifacts originating from one or a few distinct sources or a limited volume of space were removed. In addition, other artifacts characterized by a particular temporal pattern such as exponential decay were removed using an independent component analysis (ICA) algorithm.

The advantages of the ICA algorithm can be viewed in the literature (Onton, Westerfield, Townsend, & Makeig, 2006), the artifacts can be separated as independent components, and the other features of the original EEG signal can be effectively retained. Other artifacts, such as eye movement, muscle artifacts, and heart beats, could be also removed by ICA correction. The EEG data were segmented into 2 s blocks. Finally, average Fourier cross‐spectral matrices were computed for the frequency bands, including delta (0.5–3.5 Hz), theta (4–7.5 Hz), alpha1 (8–10 Hz), alpha2 (10–12 Hz), beta1 (13–18 Hz), beta2 (18.5–21 Hz), beta3 (21.5–30 Hz), gamma 1 (30.5–44 Hz), and gamma 2 (55–100 Hz).

### 2.7 Data analysis

#### 2.7.1 Source localization

Standardized low‐resolution brain electromagnetic tomography (sLORETA) was used to explore the activity of brain areas in three groups and find any abnormal activity of brain areas in acute and chronic tinnitus patients. After mean reference transformation was applied, the sLORETA algorithm was used to calculate neuronal activity as current density (A/m^2^) (Pascual‐Marqui, 2002). The LORETA‐Key software was used to investigate solution space and lead field matrix. sLORETA is a method employed to resolve the EEG inverse problem and localize the sources of EEG activity using a three‐shell spherical model (skin, skull, cortex) registered to the Talairach human brain atlas provided by the Montreal Neurological Institute (MNI) (Dümpelmann, Ball, & Schulze‐Bonhage, 2012). The software revisited realistic electrode coordinates (Jurcak, Tsuzuki, & Dan, 2007) and the lead field (Fuchs, Kastner, Wagner, et al., 2002), applying the boundary element on the MMI‐152 (Mazziotta et al., 2001). The solution space had a total of 6,239 voxels at 5‐mm spatial resolution (Lancaster et al., 2015).

#### 2.7.2 Functional connectivity measurements

When two brain areas oscillate coherently with a phase lag, cross talk can be interpreted as information sharing by axonal transmission. Phase synchronization was defined as synchronization of the phases of two coupled neural oscillatory activities, that is, the phase difference of two activities does not change with time, and there is a fixed phase difference. The linear (i.e., coherence) and nonlinear dependence (i.e., phase synchronization) between the multivariate time series were measured (Pascual‐Marqui, 2007). The results were expressed as the sum of lagged dependence and instantaneous dependence. The measures were non‐negative, and took the value zero only when the result showed independence, that is, lagged, instantaneous, or both. In the meantime, the measures were defined in the following frequency domains: delta (0.5–3.5 Hz), theta (4–7.5 Hz), alpha1 (8–10 Hz), alpha2 (10–12 Hz), beta1 (13–18 Hz), beta2 (18.5–21 Hz), beta3 (21.5–30 Hz), gamma 1 (30.5–44 Hz), and gamma 2 (55–100 Hz).

Based on this principle, the lagged linear (nonlinear) connectivity was calculated. After filtering the EEG data, the instantaneous phase was calculated using the Hilbert transform. More precisely, the data was decomposed into a limited number of cosine and sine waves at the Fourier frequencies using the discrete Fourier transform. sLORETA was applied to extract current density for regions of interest (ROI) over time. Power in all voxels was normalized to a power of 1 and log transformed at each time point. As a result, ROI values reflect the log transformed fraction of total power across all voxels for specific frequencies. ROI was confirmed based on a previous study, as shown in Table 1 (Carpenterthompson, Schmidt, & Husain, 2015).

**TABLE 1:** The regions of interest in this study

Regions of interest	BA	Centroid voxel^a^		
		*X*	*y*	*z*
Auditory cortices	41L	−46	−29	10
	41R	47	−29	10
	42L	−62	−23	12
	42R	63	−24	12
	21L	−57	−18	−15
	21R	58	−17	−15
	22L	−56	−25	5
	22R	56	−22	3
Insula	13L	−39	−8	9
	13R	40	−7	9
Dorsal anterior cingulate cortex	24L	−8	2	36
	24R	7	1	36
Pregenual anterior cingulate cortex	32L	−9	29	21
	32R	8	30	20
Subgenual anterior cingulate cortex	25L	−8	18	−17
	25R	5	14	−14
Posterior cingulate cortex	31L	−11	−50	32
	31R	9	−48	33
Parahippocampus	27L	−19	−33	−4
	27R	18	−33	−4
	29L	−7	−50	7
	29R	6	−50	8
Orbitofrontal cortex	10L	−22	54	9
	10R	22	54	9
	11L	−18	43	−17
	11R	19	43	−17
Precuneus	7L	−17	−63	50
	7R	15	−63	49

Abbreviations: BA, Brodmann area; L, left; R, right.

^a^ Coordinates are described in MNI coordinates.

### 2.8 Statistical analysis

A non‐parametric statistical analysis of LORETA‐KEY images (statistical non‐parametric mapping; SnPM) was used to compare differences in resting‐state EEG activity between the patient groups using LORETA‐KEY's built‐in voxel‐wise randomization tests (5,000 permutations) and a *t* statistic for independent groups with a threshold of *p* < .01 (corrected for multiple comparison). A correction for multiple comparisons in SnPM using random permutation has been shown to yield similar results with those acquired from a statistical parametric mapping approach using a general linear model with multiple comparisons corrections. For lagged connectivity differences, we compared differences between the chronic tinnitus and healthy control groups, between the chronic tinnitus and acute tinnitus groups and between the acute tinnitus and healthy control groups for each contrast using the *t* statistic for independent groups with a corrected threshold of *p* < .05. The significance threshold was based on a permutation test with 5,000 permutations.

## 3 RESULTS

The characteristics of acute and chronic tinnitus patients were summarized in Table 2. There were no significant differences in the score of THI, hearing threshold, and tinnitus laterality between acute and tinnitus groups. Regression analysis showed that there were no significant correlations between the activity of EEG frequency bands and tinnitus pitch and intensity of acute and chronic groups separately (*p* > .05). In addition, no significant difference was found between patient groups in terms of age (F = 0.132, *df* = 2, *p* = .877) and gender (x2 = 0.052, *df* = 2, *p* = .974).

**TABLE 2:** The demographic information and tinnitus characteristics of acute and chronic tinnitus patients

	Acute tinnitus patients (*n* = 24)	Chronic tinnitus patients (*n* = 23)	*p‐*value
**Age (years)**	47.17 ± 15.22	49.09 ± 13.78	.877
**Gender (male: female)**	M: F = 9:15	M: F = 8:15	.974
**THI score**	37.83 ± 16.71	40.61 ± 26.52	.675
**Hearing thresholds (healthy side)**	17.29 ± 7.54	23.60 ± 4.76	.099
**Hearing thresholds (tinnitus side)**	40.02 ± 10.83	43.13 ± 13.19	.391

*Note:* Data are represented as mean ± *SD*.

Abbreviation: THI, tinnitus handicap inventory.

**FIGURE 1:**
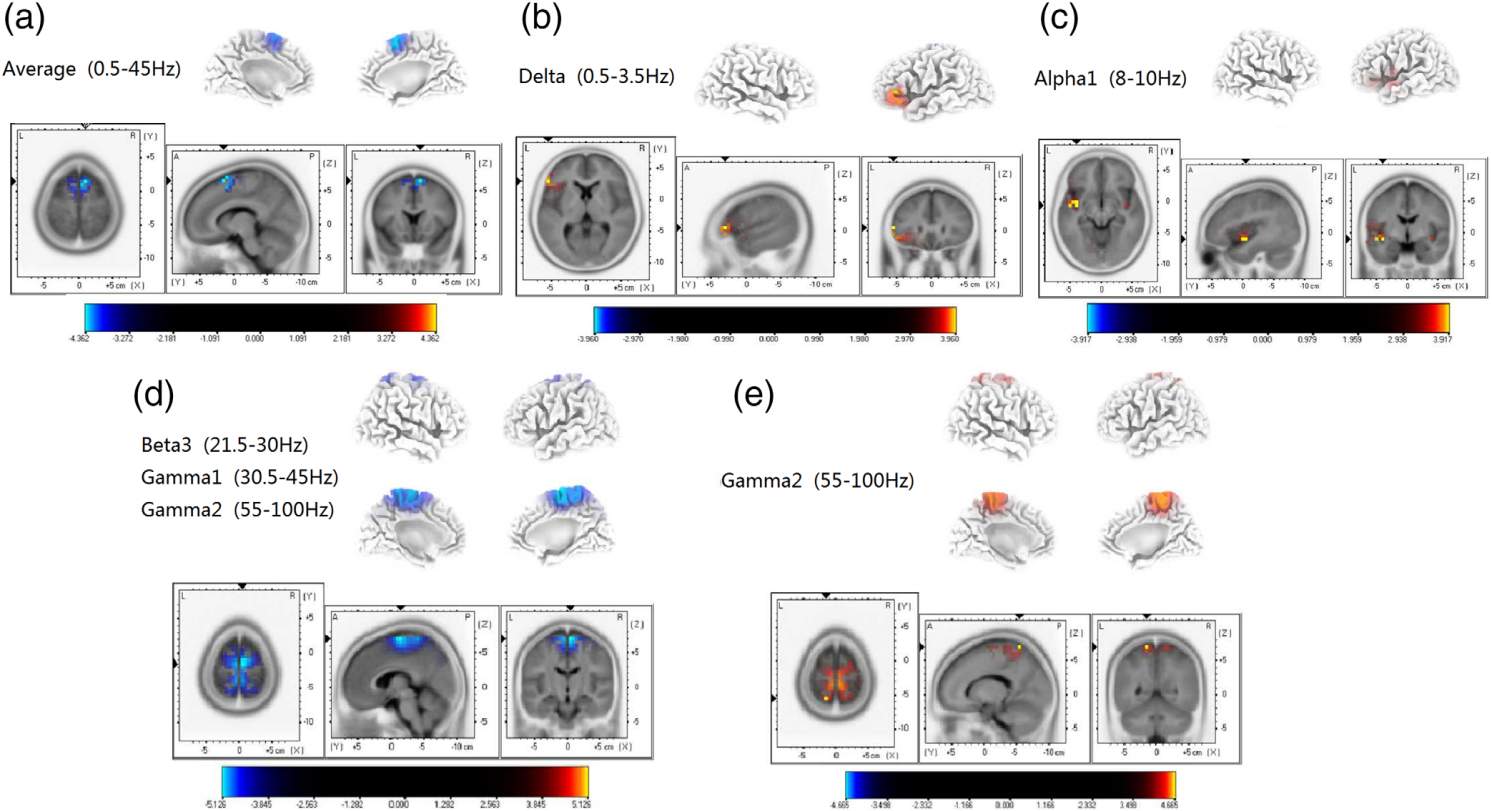
Standardized low‐resolution brain electromagnetic tomography (*p* < .05). (a) Compared with the control group, the superior frontal cortex (BA 6) of acute tinnitus patients was significantly reduced across the whole frequency band. (b) Compared with the control group, the inferior frontal gyrus (BA 47) of chronic tinnitus patients was significantly enhanced in the delta frequency band. (c) Compared with the control group, the superior temporal gyrus (BA 13) of chronic tinnitus patients was significantly enhanced in the alpha 1 frequency band. (d) Compared with the control group, the superior frontal cortex (BA 6) of chronic tinnitus patients was significantly decreased in the beta 3 and gamma frequency bands. (e) Compared with the chronic tinnitus group, the middle temporal gyrus and parietal gyrus (BA 6, BA 7) of acute tinnitus patients were significantly increased in the gamma frequency band

Acute tinnitus patients had a significant reduction in superior frontal cortex (BA 6) activity across all frequency bands when compared with the healthy subjects (see Figure 1a; *p* < .05). In contrast, compared with the healthy subjects, chronic tinnitus patients had reduced brain activity in the superior frontal cortex (BA 6) for beta 3 and gamma frequency bands. In addition, chronic tinnitus subjects had a significant increase brain activity in the inferior frontal cortex (BA 47) for the delta‐band and the superior temporal cortex (BA 13) for alpha 1 frequency band when compared with the healthy subjects (see Figure 1b–d; *p* < .05). The acute tinnitus group activity was however significantly increased in the middle frontal gyrus and the parietal gyrus (BA 6, BA 7) for gamma‐band when compared to the chronic tinnitus group (see Figure 1e; *p* < .05). No significant difference was found in other brain areas and frequencies.

**FIGURE 2:**
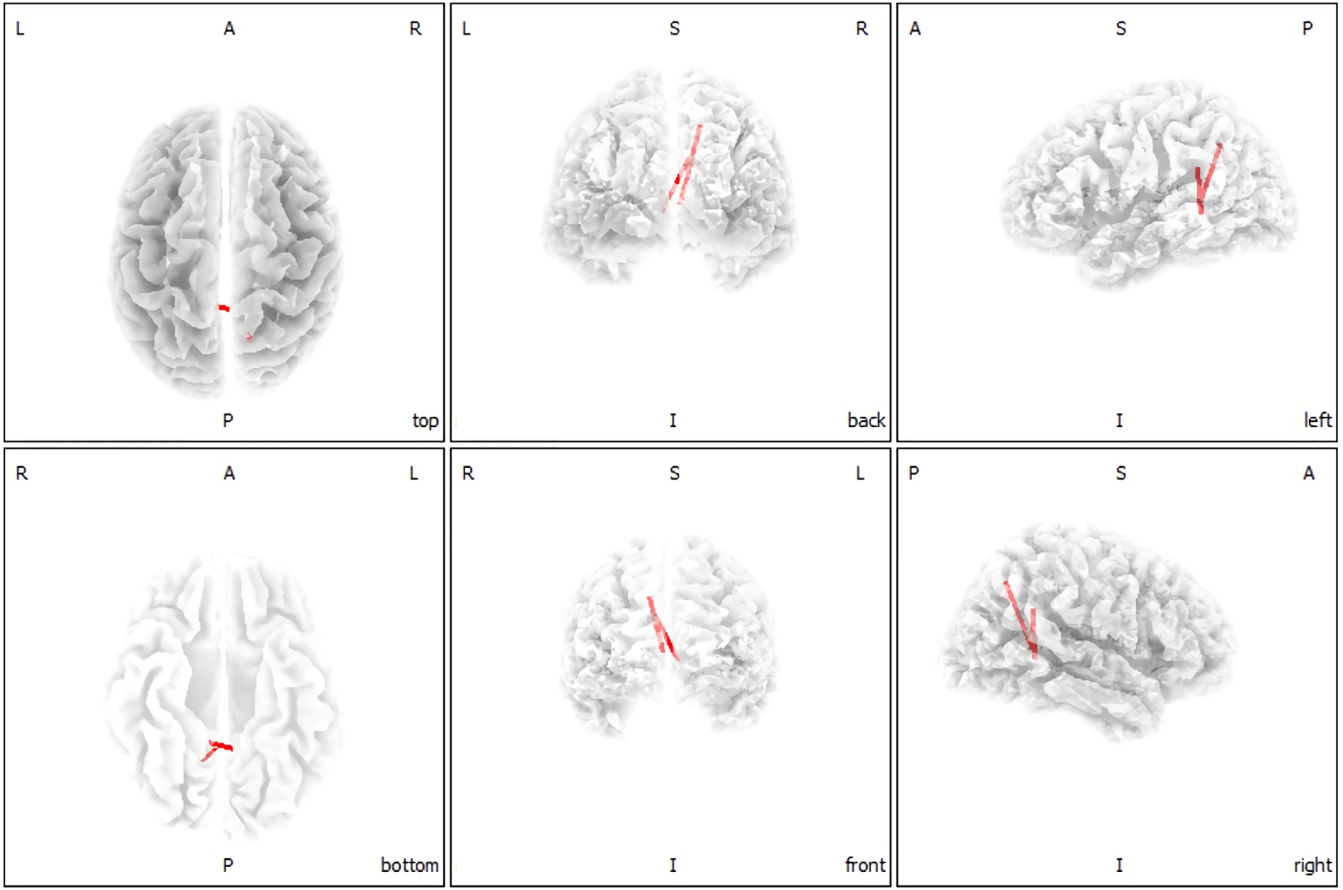
Functional connectivity in chronic tinnitus patients (*p* < .05). According to the lagged phase synchronization analysis, the significantly enhanced connections were found between the parahippocampus gyrus (BA 21) and the posterior cingulate cortex (BA 18) as well as between the parahippocampus gyrus (BA 22) and precuneus (BA 28) in chronic tinnitus patients

When compared with the healthy group, significantly increased functional connections were shown in chronic tinnitus patients between the parahippocampus gyrus (BA 21) and the posterior cingulate cortex (PCC) (BA 18) as well as between the parahippocampus gyrus (BA 22) and precuneus (BA 28) under the lagged phase synchronization analysis (see Figure 2; *p* < .05). However, no significant connectivity change was found in acute tinnitus patients when compared with healthy subjects or chronic tinnitus patients.

## 4 DISCUSSION

To our knowledge, this is the first study to identify the central processing network and neural predictors for the development of tinnitus by using source localization and functional connectivity methods. Although a number of previous studies on chronic tinnitus have been published and many pathophysiological models have been developed (Chen et al., 2017; Chen et al., 2018; Vanneste, Song, & De Ridder, 2018; Vanneste, To, & De Ridder, 2019), few studies have investigated central plasticity in acute tinnitus (Cai et al., 2019; Cai et al., 2020), especially the dynamic changes to neural processing in the transition period from acute to chronic tinnitus. This issue is crucial to clarify central neural characteristics of acute and chronic tinnitus, which is important in identifying neural predictors for the development of tinnitus and prevent transition from acute to chronic. A major finding of this study was that both acute and chronic tinnitus patients had aberrant neural activity in the superior frontal cortex when compared to the control group. Respectively, increasing gamma‐band activity in the middle frontal gyrus and the parietal gyrus was observed for the acute tinnitus group. In contrast, an increase in the inferior frontal cortex for the delta‐band and the superior temporal cortex for alpha 1 frequency band was detected in patients with chronic tinnitus. Functional connectivity analysis showed the chronic tinnitus group to have increased connections between the parahippocampus gyrus and other nonauditory areas (PPC and precuneus) when compared with the control group. Abnormalities within the frontal, parietal, and temporal cortex identify the changed neural target in the dynamic development of tinnitus. In addition, aberrant connections imply that the parahippocampus gyrus is likely a major hub in the transition from acute to chronic tinnitus.

### 4.1 Reduction of activity in superior frontal cortex in both acute and chronic tinnitus

A significant reduction of activity in the superior frontal cortex (SFC) could be detected in patients with either acute or chronic tinnitus. The SFC has been regarded as the integrative hub of tinnitus that organizes efferent impulses to ensure coordination of the central nervous system (Chen et al., 2016; Mathew et al., 2007). The SFC is related to various cognitive and executive control tasks. Li et al. (Li et al., 2013) divided the human SFC into anteromedial (SFCam), dorsolateral (SFCdl) and posterior (SFCp) subregions based on diffusion tensor imaging. Furthermore, it has been shown that the SFCam and SFCdl are anatomically and functionally correlated with the executive control network (ECN) and default mode network (DMN) and the SFCp is correlated with sensorimotor‐related brain areas. Thus, reduced SFC activity might suggest decreased use of the ECN in tinnitus patients, possibly related to the attention paid to the phantom noise and its influence on their cognitive control. This is also in line with a dysfunctional top‐down noise‐canceling mechanism for tinnitus. Previous studies have postulated that the frontal cortex is involved in top‐down modulation in a frequency‐specific manner (Helfrich, Huang, Wilson, & Knight, 2017; Vanneste, Alsalman, & De Ridder, 2019). In the context of the existing literature, the default mode alpha activity represents a functional top‐down control system that actively blocks disturbing noise in tinnitus (Joos et al., 2012). The alpha activity within the superior frontal cortex decreased only in acute tinnitus subjects, whereas alpha activity in chronic tinnitus subjects was not significantly different in comparison to the healthy controls. It might imply enhanced alpha activity during the move from acute to chronic tinnitus. Normally, alpha waves are recorded from the auditory cortex during the resting state. Thus, the altered alpha activity in the superior frontal cortex might indicate plasticity and adaptation to offset the phantom noises in the development of tinnitus.

### 4.2 Enhanced brain activity of middle frontal gyrus and parietal gyrus in acute tinnitus

Increased gamma‐band activity was found in the middle frontal gyrus (MFG) and the parietal gyrus of the acute tinnitus group when compared to the chronic tinnitus patients. The parietal gyrus is traditionally considered to be involved with attention, perceptual decision making and sensorimotor transformations (Freedman & Ibos, 2018; Sestieri, Shulman, & Corbetta, 2017). The posterior part of the parietal gyrus has functions in feature‐independent coding, enhancement of activity by attention and representation of task‐related signals. The MFG is involved in the frontoparietal attention network (Ptak, 2012). In tinnitus patients, the processing of phantom noise can cause cognitive interference. Consequently, networks that enable focusing of attention that prevent the unconscious conversion of phantom to salient noises collide (Schmidt, Akrofi, Carpenterthompson, & Husain, 2013).

Thus, tinnitus might deplete cognitive resources and compromise attending to visual tasks such as reading. Prior behavioral evidence indicates that tinnitus disrupts the allocation of attention to nonauditory stimuli (Burton et al., 2012). It has been proposed that tinnitus conflicts sufficiently with nonauditory sensory processes to alter concentration, thereby lowering accuracy on attention demanding tasks. In this research, increased beta 3 and gamma activity found in the frontal and parietal regions could imply that patients with acute tinnitus might pay more attention to auditory stimuli and message processing.

### 4.3 Increased brain activity in inferior frontal cortex and superior temporal cortex in chronic tinnitus

In the patients with chronic tinnitus there was significantly enhanced low frequency (Delta and alpha bands) activity in the inferior frontal cortex (IFC) and superior temporal cortex (STC). IFG acts as an executive control component in the attention system that regulates dorsal and ventral attention networks (Sebastian et al., 2016). The STC is the center of the primary auditory cortex and related to auditory perception. The STC is considered to be an important multisensory functional brain region, which integrates visual, auditory and language information (Zevin, 2009). It has been argued that low‐frequency neuronal oscillations might serve as a cortical mechanism for sensory selection, attention allocation, and evidence updating (Schroeder & Lakatos, 2009). Analogous to deep‐sleep states or neuronal dysfunction, low frequency oscillations might hinder active processing in patients with chronic tinnitus. Because of their control of neuronal excitability and sensory processing, low‐frequency oscillations could play a role in attention selection. Previous studies have shown a link between low neuronal oscillations in the frontal regions and conscious perception (Mathew et al., 2007) and frontal delta activity with visual perception (Helfrich et al., 2017). In summary, delta and alpha activity observed in the frontal and temporal cortex might reflect an involvement of emotion, perception, and attention in the transition from acute to chronic tinnitus.

### 4.4 Connectivity between the parahippocampus gyrus and other nonauditory areas

Significantly increased connections between the parahippocampus gyrus and other nonauditory areas (PCC and precuneus) were found only when the chronic tinnitus group was compared to the control group. Based on a hypothesis (De Ridder et al., 2006), increased activity in parahippocampal areas might reflect the constant updating of tinnitus perception, preventing habituation by means of its sensory gating function. The cells in the human hippocampus and parahippocampal areas increase their firing in response to new stimuli. The number of cells responding to stimulation in the parahippocampal region decreased sharply, suggesting rapid adaptation. In contrast to the rapid auditory habituation of the parahippocampal region, a large number of neurons in the hippocampus showed inhibitory responses (Viskontas, Knowlton, Steinmetz, & Fried, 2006). Dysfunction of this mechanism has been hypothesized to explain complex auditory experiences such as tinnitus perception (Diederen et al., 2010). In addition the parahippocampus has also been hypothesized to contribute to auditory memory, sending information from the hippocampus to the relative areas (Leaver et al., 2016). Vanneste et al. (Vanneste & De Ridder, 2016) explored the mechanisms of different amounts of hearing loss in tinnitus and found tinnitus with little or no hearing loss to be more related to auditory cortex activity, whereas tinnitus with more severe hearing loss seemed to be related to parahippocampal activity. This supports the idea of a compensation mechanism where, as tinnitus can be seen to result from persistent parahippocampal activity, to fill the missing auditory information it constantly sends stored auditory information from the hippocampus to the auditory and other nonauditory areas.

The PCC and precuneus are important structures of the default mode network DMN, which is most active at rest and shows reduced activity when entering a task‐based state involving attention and goal‐directed behavior (Mantini, Perrucci, Gratta, Romani, & Corbetta, 2007; Raichle et al., 2001). Another study (Husain & Schmidt, 2014) has shown decreased connectivity in the (DMN), involving the precuneus and PCC in tinnitus patients. Previous fMRI studies found abnormal functional connectivity within the DMN related to tinnitus distress (Burton et al., 2012; Schmidt et al., 2013). A positive association has also been found between THI score and beta values of the posterior cingulate as well as the precuneus region (Maudoux et al., 2012). Brain regions in the PCC/precuneus (Krick, Argstatter, Grapp, Plinkert, & Reith, 2017) and parahippocampus (Kim et al., 2016) were also reported to have significantly altered functional activity with improvement of distress after tinnitus therapy. In addition, frontal brain areas (SFG, MFG), the PCC, parahippocampal gyrus and precuneus are included within the frontal–parietal‐limbic network, which has been regarded as a specific distress network in tinnitus and more active in tinnitus patients with serious distress and high THI scores (Golm, Schmidtsamoa, Dechent, & Kronerherwig, 2013; Husain, 2016; Husain & Schmidt, 2014). Our findings support the view that alterations within these areas in the transition from acute to chronic tinnitus may also be a reflection of the distress that accompanies the development of tinnitus. We suggest that connectivity between parahippocampus gyrus and other nonauditory cortex may play a crucial role in the development and maintenance of chronic tinnitus.

Limitations must be acknowledged in this study. This is a cross‐sectional study with a limited sample size. Due to patient compliance, it is difficult to longitudinally analyze the move from acute to chronic onset with tinnitus subjects. In addition, we mainly investigated differences in cortical activity and connectivity between acute and chronic tinnitus. Although there were mild to moderate hearing loss on the tinnitus side, no significant differences were found in the hearing thresholds and laterality of tinnitus between two groups, hearing threshold and tinnitus laterality effect on brain network were not further explored in the present study. It would be interested to explore the influence of various factors on the central neural alterations in patients with tinnitus by categorizing them on the basis of the tinnitus characteristics (e.g., tinnitus laterality and hearing thresholds).

## 5 CONCLUSION

With increase in tinnitus duration neural activity in the frontal, parietal and temporal cortices changed continuously and connectivity between the parahippocampus gyrus and other nonauditory areas appeared gradually. This study indicates that the occurrence and development of tinnitus is a dynamic process from abnormal local neural activity to abnormal connectivity in multifunctional brain networks. Particularly, the present results suggest that alterations of local brain activity and connections between the parahippocampus gyrus and other nonauditory areas may predict the transition from acute to chronic tinnitus and the parahippocampus gyrus appears a key hub in the central mechanism of tinnitus.

## CONFLICT OF INTERESTS

The authors declare that the research was conducted in the absence of any commercial or financial relationships that could be construed as a potential conflict of interest.

## Data Availability

There is no other available data. However, the data related to the results of the study can be obtained by emailing caiyx25@mail.sysu.edu.cn.
